# Treatment of Erectile Tumors

**Published:** 1878-04

**Authors:** 


### EXCERPTA.

TREATMENT OF ERECTILE TUMORS.—Dr. Notta, having had an opportunity of treating a large number of erectile tumors, gives his ideas as to the various methods available for this purpose in *L'Annee Medicale* (1877, No. 12). He divides them into two classes, cutaneous and subcutaneous. When called upon to treat the superficial variety in children who have not been vaccinated, vaccination offers perhaps the best method of treatment. A number of needles are dipped into vaccine matter to the extent of six millimetres, and this is allowed to dry. They are then thrust into the tumor to the depth of four millimetres, and at a distance of three millimetres from one another. Care should be taken to insert a row along the line of separation between the tumor and the healthy skin. After remaining in place about an hour, the needles are withdrawn, and some days later the punctured locality becomes the seat of a confluent vaccinal eruption. A month later, the eruption has entirely disappeared, or if there still remains a point here and there, the application of a white-hot needle will finish the operation.

When for any reason vaccination cannot be practiced, the double or multiple-crossed ligature may be employed with success, according to the method of Rigal de Gaillac. The tumor is strangulated by this procedure; the ligatures drop out at the end of four to eight days, leaving a granulating ulcer, which heals slowly. As regards the treatment of subcutaneous erectile tumors, one very good method is that by strangulation with vaccine-impregnated ligatures; other methods are the white-hot needle or the injection of the perchloride of iron. Dr. Notta continues his observations in a subsequent number of *L'Annee Medicale*, and gives other methods of treatment.—*Philadelphia Medical Times.*

